# The effects of active rehabilitation on symptoms associated with tau pathology: An umbrella review. Implications for chronic traumatic encephalopathy symptom management

**DOI:** 10.1371/journal.pone.0271213

**Published:** 2022-07-21

**Authors:** Rachael Hearn, James Selfe, Maria I. Cordero, Nick Dobbin

**Affiliations:** 1 Faculty of Health, Department of Health Professions, Psychology, & Social Care, Manchester Metropolitan University, Manchester, United Kingdom; 2 Faculty of Health, Department of Psychology, Psychology, & Social Care, Manchester Metropolitan University, Manchester, United Kingdom; University of Florida, UNITED STATES

## Abstract

**Objective:**

This review sought to address an evidence gap and lay a foundation for future Chronic Traumatic Encephalopathy (CTE) management studies by evaluating and appraising the literature which reports the effect that active rehabilitation has on other tauopathies, a group of conditions with hyperphosphorylation and aggregation of tau protein that can lead to neurodegeneration.

**Design:**

Umbrella review.

**Data source:**

Meta-analyses and systematic reviews were identified using CINAHL, Medline, Cochrane, Web of Science, PubMed, and SPORTDiscus.

**Eligibility:**

Systematic review or meta-analyses that examine the effect active rehabilitation has on outcome measures of symptoms associated with CTE. Studies with men and women diagnosed with Alzheimer’s disease, Parkinson’s disease, Lewy Body dementia, Frontotemporal degeneration/dementia or Corticobasal degeneration. All types of active rehabilitation were included. Control group was usual care, no intervention, or light-intensity physical activity.

**Results:**

Twelve reviews were included. A large pooled standardized mean difference (SMD) was observed for balance (SMD = 0.88, P<0.001) and motor function (SMD = 0.83, *P*<0.001). A moderate pooled SMD was observed for cognitive function (SMD = 0.66, *P<*0.116). A small pooled SMD was observed for mobility (SMD = 0.45, *P* = 0.002). A trivial pooled SMD was observed for gait speed/velocity (SMD = 0.11, *P* = 0.372). No findings for mood/behavioral symptoms. All pooled effects demonstrated substantial to considerable heterogeneity (74.3% to 91.9%, P<0.001).

**Conclusions:**

A positive effect of active rehabilitation was observed in patients with tau pathologies suffering from motor, vestibular and cognitive impairments supporting the use of active rehabilitation for CTE management; however, the findings need to be considered with caution given the limited research in some of the tau pathologies, large between-study heterogeneity and wide 95% prediction intervals.

## Introduction

Phosphorylated microtubule-associated tau proteins are a necessary component of neural health and functioning; however, tau proteins also have the potential to serve as a catalyst for neurodegeneration in a group of pathologies collectively termed tauopathies [1–3]. Tau stabilizes those microtubules which provide shape and structure to neural axons, dendrites and synapses. Tau has also been found to aid in axonal transport, synaptic transmission, cytoskeletal regulation and proteostasis [2–4]. Tau has a reversible hyperphosphorylation capability which provides neural protection and regulation. Subsequently, the development of pathogenic tau formulation has been associated with irreversible hyperphosphorylation and the disruption of microtubule stability [4]. It remains unclear what order this occurs in or what the specific pathophysiology is [4], but the release of tau and translocation to synapses may lead to further spread of pathogenic tau [1,2]. Accumulation of pathogenic tau can disrupt neural connectivity and synaptic function, eventually leading to neural cell death and atrophy of several brain regions characteristic of diseases such as Alzheimer’s disease (AD), Corticobasal degeneration (CBD), Frontotemporal degeneration/dementia (FTD), Lewy Body disease (LBD) and Parkinson’s disease (PD). Factors such as genetics or metabolic syndromes can trigger a pathogenic tau formation. Exposure to brain injury has also been found to result in neurodegenerative tauopathy, termed Chronic Traumatic Encephalopathy (CTE) [1,3,4].

An observed relationship between contact sport participation and compromised brain health dates back to 1928 when Dr. Harrison Martland published the first description of a syndrome known as ‘Punch Drunk’ [5–8]. Dr Martland noted a clinical pattern of cognitive, mood, motor and behavioral changes in boxers and believed that exposure to repetitive head impacts was the major contributing factor for developing such a syndrome [5]. Formally established in 2015, CTE is a tauopathy defined by its distinct irregular spatial pattern of abnormal tau accumulation in neurons and astroglia around small blood vessels at the base of sulci of the cortex [7]. Specific mechanisms, including the progressive nature of CTE and additional factors contributing to the pathological development, remain largely unestablished [6,8]. Kriegel and colleagues [9] proposed the axonal and microvascular injuries which lead to persistent neuroinflammation and metabolic disruption that are sustained due to mTBI may trigger another ‘pathological cascade’ that leads to the development of CTE in at-risk individuals. It’s suggested that the abnormal tau proteins accumulate at the area of initial injury, with chronic neuroinflammation further exacerbating the neurodegeneration and eventual development of those characteristics unique of CTE [9]. Other research groups have proposed that CTE is simply one component to be considered among a much broader evaluation of long-term consequences regarding a history of exposure to contact sport and mTBI, with some suggesting the potential for an increased risk of developing neurodegenerative disease/dementia [10–12] and others suggesting the link may simply indicate an earlier onset of neurodegeneration/dementia in those already considered at-risk [13,14].

To deepen our understanding of the pathology, it is vital that research is conducted to better establish epidemiology, risk factors, and diagnostic tools to aid in the prevention and treatment of CTE. While CTE can only be diagnosed post-mortem [5,7], based on the significant increased risk of developing neurodegenerative disease/dementia that former contact sport athletes face [15–17], it can be assumed that a significant number of former athletes are currently living with or at risk of soon developing symptoms of neurodegeneration, some of which can likely be attributed to the presence of CTE. Currently, no treatment has been validated by clinical trials to be used for the management of CTE [5]. Experts have suggested looking at other similar neurodegenerative conditions [6]. Tau targeting therapies intended to modify disease progression in tau pathologies are still under investigation, with symptomatic management remaining the primary intervention strategy for tauopathies. A combination of pharmacological therapy, physical therapy and psychotherapy may be beneficial for managing cognitive dysfunction, motor dysfunction and changes in mood and behavior associated with tauopathies [1]. Active rehabilitation, defined as an exercise-based rehabilitation program designed to improve functioning, is one therapy that has been suggested [5]. Active rehabilitation has the ability to enhance neuroprotection, neurogenesis, neuroplasticity, angiogenesis, cerebral perfusion, vasoreactivity, blood-brain barrier permeability and ATP production [18–21].

To the best of the authors’ knowledge, no review to date has been conducted to determine if active rehabilitation is a management tool that can be applied broadly to patients suffering from tau pathology. Further, no studies have been published that establish an evidence-based intervention strategy precisely intended for the symptoms or processes of suspected CTE. Therefore, the purpose of this umbrella review is to establish the potential for active rehabilitation as an intervention strategy for the management of suspected-CTE by evaluating and appraising the evidence regarding the effect that active rehabilitation has on other tauopathies. Unlike a systematic review which seeks to synthesize evidence regarding a specific area of research, an umbrella review examines and summarizes the literature by comparing/contrasting findings of different research syntheses, considering factors such as effect size, consistency and quality. This can include an examination across a broad range of conditions, interventions and outcomes [22–24]. Undertaking a review of this type will establish whether active rehabilitation is a successful management strategy across a range of tauopathies, subsequently addressing an evidence gap within the field of CTE interventions.

## Methods

This umbrella review was performed following guidelines set out by Armataris et al. [22] in association with the Joanna Briggs Institute (JBI) [23].

### Search strategy

A computerized systematic search of CINAHL, Medline, Cochrane, Web of Science, PubMed, and SPORTDiscus (all years to 09/10/2020) using the search syntax outlined in Table 1 was conducted in October 2020.

**Table 1:** Search and PICO.

Search syntax	(disease OR disorder OR symptom* OR dementia OR *degenerat*) AND (Alzheimer OR Parkinson OR “Lewy body” OR frontotemporal OR corticobasal) AND (therapy OR intervention OR treatment OR rehabilitation) AND (exercise OR "physical activity" OR "resistance training" OR "aerobic exercise" OR "balance training" OR walking OR sport OR yoga OR pilates) AND ("systematic review")	
Population	Men and women diagnosed with Alzheimer’s disease, Parkinson’s disease, Lewy Body dementia, Frontotemporal degeneration/dementia, Corticobasal degeneration	
Intervention	Active rehabilitation of any type. Interventions that combined active rehabilitation with other techniques (e.g., pharmacological treatment + exercise) were excluded.	
Comparator	Usual care, no intervention, light-intensity physical activity [25]	
Outcome	Outcome measures which report on shared symptoms associated with CTE (listed below)	
	**Behavioral**	**Cognitive**
	Physical violence	Impaired memory
	Inappropriate behavior	Attention/concentration
	Verbal violence	Executive dysfunction
	Explosivity/short fuse	Dysgraphia*
	Loss of control/disinhibition	**Motor**
	Personality changes	Dysarthria*
	Impulsivity	Ataxia*
	Paranoid delusions	Parkinsonism*
	**Mood**	Muscle tremor
	Depression	Masked facies*
	Anxiety	Muscle rigidity
	Aggression	**Vestibular/Ocular**
	Irritability	Balance
	Mood swings	Visuospatial difficulty
	Apathy	Blurred/double vision
	Insomnia	Dizziness

Information on literature search and selection criteria [5,26]

*Ataxia: Slurred speech, incoordination. Dysarthria: Speech difficulty. Dysgraphia: Impaired writing ability. Masked facies: Loss of facial expression. Parkinsonism: Movement abnormalities.

### Eligibility criteria

Studies were included if the full-text was available and were peer-reviewed systematic reviews or meta-analyses that examined the efficacy of active rehabilitation in the management of common neurodegenerative diseases with tau aggregation. Only reviews written in English and with data presented in a way that could be extracted by the authors were included. Further inclusion criteria were defined according to the Participant-Intervention-Comparison-Outcome (PICO) process, included in Table 1.

Two authors (RH and ND) independently screened the title, abstract, and full text for eligibility. If disagreement between reviewers occurred, a consensus eligibility method was used. A third reviewer was not needed as there was no circumstance in which a consensus could not be reached.

### Quality evaluation

Two authors (RH and ND) independently assessed the methodological quality of the included systematic reviews and meta-analyses using the JBI Critical Appraisal Checklist for Systematic Reviews and Research Syntheses [23]. Eleven factors (detailed in Fig 2) were assessed for appropriateness or adequacy in relation to objectives, including inclusion criteria, search strategy, appraisal strategies, analysis strategies, and conclusions drawn. A point was given for each component addressed. Scores range from 0–11 points. Higher scores indicate higher levels of methodological quality. Discrepancies between reviewers were resolved through discussion and a consensus was reached without the need of a third reviewer.

### Data extraction

Data was extracted independently by primary author (RH) and checked by a second author (ND) using the JBI Data Extraction Form for Review for Systematic Reviews and Research Syntheses [23]. This included recording information on author, year of publication, country of origin, objectives, results, appraisal, appraisal instruments, appraisal rating, and other relevant information on the primary level studies included in the review.

In addition to completing the JBI extraction checklist for each included review, the standardized mean difference (SMD), 95% confidence intervals (CI), and number of studies included for all eligible meta-analyses were extracted. If a pooled effect was not available for a given study, a random effects model was run to calculate the missing values using the available mean, standard deviation, and number of participants for the intervention and control groups. This model was conducted using the metafor function in R (R Studio, Version 1.2.1335).

### Statistical analysis

The results of the data syntheses were grouped by clinical features, as illustrated in Table 1 [5,26]. The magnitude of the effect of the intervention was assessed as a standardized mean difference (SMD) and more precisely, Hedges g. SMD values were classified according to Cohen’s definition, with effect values interpreted as: <0.20, trivial; 0.20–0.50, small; 0.51–0.80, moderate; >0.80, large [27]. Variability of the intervention effect was assessed by 95%CI and a 95% prediction interval (95%PI) was derived. The 95%PI was calculated using the number of included studies, SMD, the upper limits of the 95%CI and tau squared. For each group (outcome measures), a pooled SMD (Hedges g) and 95% CI was calculated using a random effects model using the metafor package in R (R Studio, Version 1.2.1335). Heterogeneity (I^2^) was classified according to the Cochrane’s definition [28], with 0–40% considered likely not important, 30–60% representing a moderate level of heterogeneity, 50–90% representing a substantial heterogeneity, and 75–100% noting considerable levels. A decision on whether heterogeneity was significant or not was based on the Q statistic.

## Results

### Search results

**Fig 1:**
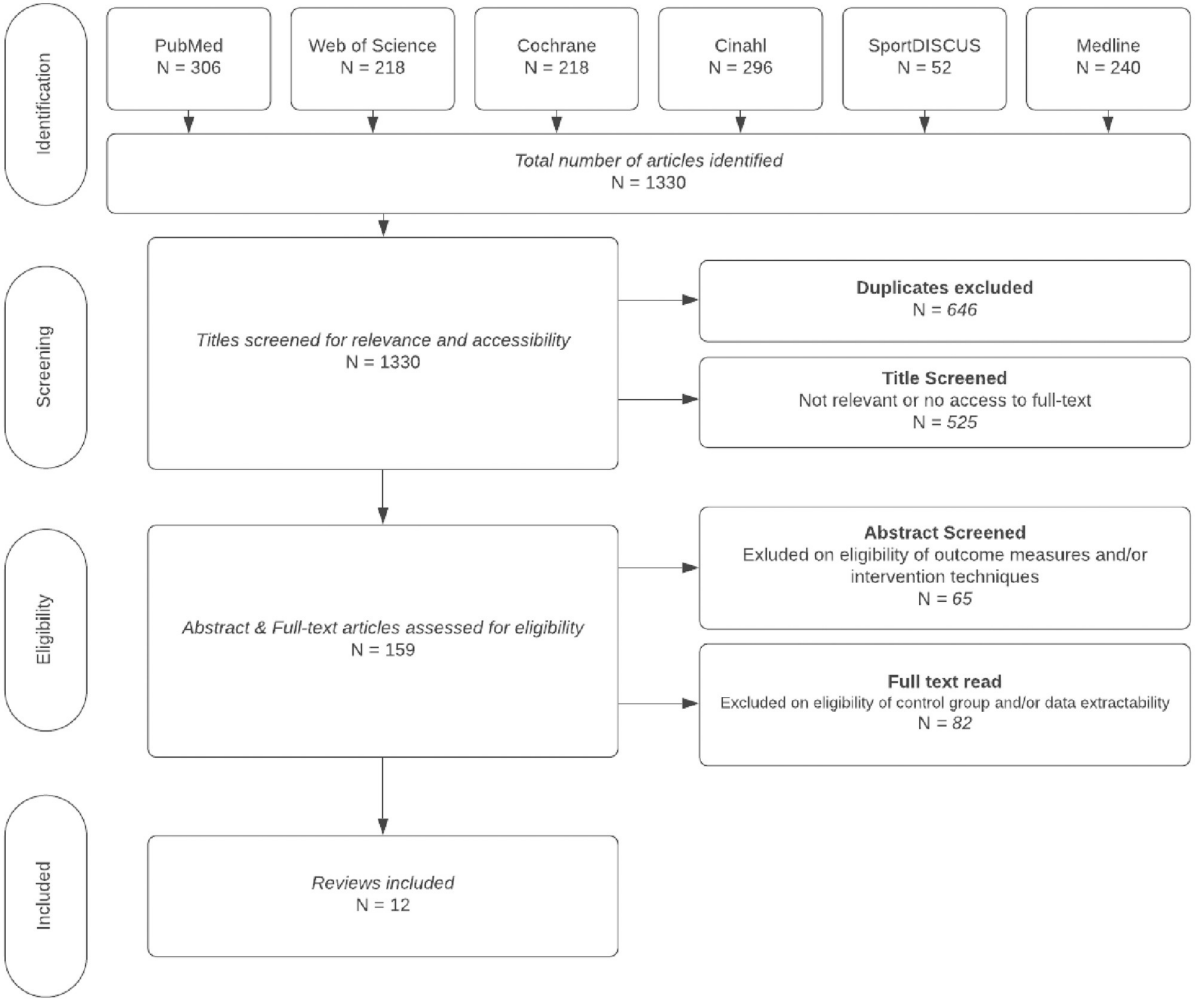
PRISMA. PRISMA flowchart indicating the study selection process.

The search identified 1,303 potential articles (Fig 1). After duplicates were removed, 774 titles were screened. From those remaining, 629 were excluded based on relevancy or access to the article, leaving 145 abstracts to be screened for eligibility. Fifty-one abstracts did not indicate relevant outcome measures and/or intervention techniques as defined by the criteria set out in Table 1, leaving ninety-four articles. Eighty-two articles did not include extractable data and/or ‘true’ control groups; therefore, a total of twelve articles were included for quality evaluation and data synthesis. Characteristics of each study can be found in Table 2.

**Table 2:** Study characteristics.

Study	Primary Studies	Participant Characteristics	Intervention & Control	Outcomes	Significance	Appraisal
**Allen (2011) [40]**	16 RCT, qRCT	*n* = 747 PD Mild-moderate severity Age range: 62.9+/- 11.9 to 75.8+/- 4.2	Intervention: exercise (aerobic, resistance, Tai Chi, dance) Control: no intervention, TAU, education classes, flexibility exercise	**Balance** (BBS, single leg stand time, tandem stance) **Functional mobility** (TUG, sit to stand time, turning time, step length, cadence) **Gait** (gait time, gait velocity)	Significant positive effect on balance. Non-significant positive effect on functional mobility and gait. Heterogeneity: Balance: 0–72% Turning time: 0% Functional mobility: 0–37% Gait: 6% (Dependent on outcome measure)	Cochrane risk of bias tool Mod-high quality: 7 Insufficient info: 8
**Alves Da Rocha (2015)** [29]**	2 RCT	*n* NR PD Age NR	Intervention: dance Control: no intervention	**Balance** (BBS) **Motor function** (UPDRS III) **Gait** (6mWT)	Positive effect on for gait, balance and motor function Heterogeneity: Balance: NA Motor function: 97% Gait: 91%	PEDro scale Good: 1 Fair: 1
**Sharp & Hewitt (2014)** [34]**	2 RCT	*n* = 137 PD H&Y mean: 2.1, 2.6 Mean age: 66.6, 69.9	Intervention: dance Control: no intervention	**Balance** (BBS) **Motor function** (UPDRS III) **Functional mobility** (FoG) **Gait** (6mWT, gait velocity)	Significant positive effect on motor function, balance, gait velocity. No effect on functional mobility. Heterogeneity Balance: 0% Motor function: 0% Functional mobility: 0% Gait: 0–45% (Dependent on outcome measure)	Cochrane Collaborations risk of bias assessment tool Individual reports not available.
**Winser (2018)** [37]**	2 RCT	*n* = 96 PD Age NR	Intervention: Tai Chi Control: no intervention, other active treatments	**Functional mobility** (TUG)	Significant positive effect Heterogeneity NR	PEDro: High GRADE: High
**Ströhle (2015)** [35]**	4 RCT	*n* = 119 AD MMSE scores: 13–22 Age NR	Intervention: exercise treatment Control: TAU, daily organized activities, home safety assessment sessions	**Global cognitive function** (ADAS-cog, ERFC, MMSE)	Moderate to strong effects Heterogeneity: 61.6%	Cochrane Collaboration’s tool for assessing risk of bias Synthesis NR
**Cai (2017)** [30]**	13 RCT	*n* = 958 AD MMSE scores 5.8–22 (2 NR) Age: 72.4–81.8	Intervention: aerobic, resistance, combined Control: no exercise	**Global cognitive function** (MMSE, CDT, FACS)	Positive overall random effect on cognitive function Heterogeneity: 77%	Downs and Black Quality Index 5: good 7: moderate 1: poor
**Santos Delabary (2017)** [33]**	2 RCT	*n* = 83 PD H&Y stages 1–4 Age range: 66.5+/-2.8 to 69.3+/-1.9	Intervention: dance classes Control: no intervention	**Motor function** (UPDRS III) **Functional mobility** (FoG) **Gait** (6mWT, gait velocity—forward, backward)	Significant positive effect on motor function. Non-significant positive effect on gait and functional mobility. Heterogeneity: Motor function: 0% Functional mobility: 0% Gait: 0%	Cochrane criteria Synthesis NR
**Kwok & Chan (2016) [32]**	6 RCT, 4 CCT	*n* NR Range: 13–80 PD Severity: mild-moderate Mean age: 60.8–74.9	Intervention: Mind & body, yoga, Tai Chi, dance Control: no intervention, placebo, waitlist, usual care, non-exercise control	**Balance** (BBS) **Motor function** (UPDRS III) **Functional mobility** (TUG) **Gait** (6mWT)	Large significant effect on motor symptoms, balance and postural instability. Moderate significant effect on functional mobility Heterogeneity Balance: 0%-89% Motor function: 0–60% Functional mobility: NA-95% Gait: NA-0% (Dependent on intervention mode)	Effective Public Health Practice Project 1: strong 5: moderate 4: weak
**Flynn (2019)** [31]**	11 RCT, 1 qRCT	*n* = 1,496 PD Mild-moderate severity Mean age: 60–72	Intervention: home-based exercise Control: TAU, placebo	**Balance** (SPPB, BBS, miniBESTest) **Gait** (time taken to walk, preferred gait speed, fast gait speed, TUG, FGA, 180 deg. turn test)	Positive effect on balance and gait speed Heterogeneity: Balance: 0% Gait: 0%	PEDro 10: good 2: fair
**Tomlinson (2012)** [36]**	20 RCT	*n* = 1,570 PD H&Y stages 2.1–2.6 Mean age: 65–69	Intervention: physiotherapy, exercise, treadmill, dance, martial arts Control: no intervention, placebo	**Balance** (BBS) **Motor function** (UPDRS III) **Gait** (speed, TUG)	Significant positive effect on balance, gait and motor function. Heterogeneity Balance: NA-75% Motor function: 0%-87% Functional mobility: 0%-48% Gait: 0%-34% (Dependent on intervention mode)	Synthesis NR
**Yang (2014)** [39]**	4 RCT, 1 nRCT	*n* = 190 PD H&Y stages 1.5–4 Mean age: 63–69	Intervention: Tai Chi Control: placebo, no intervention, other therapies	**Balance** (BBS, 1 leg stance, tandem stance) **Motor function** (UPDRS III) **Functional mobility** (TUG) **Gait** (gait velocity, 6mWT)	Significant positive effect on balance, motor function and functional mobility. Insufficient evidence of effect on gait. Heterogeneity Balance: 0–68% Motor function: 57% Functional mobility: 0% Gait: 0% (Dependent on outcome measure)	Cochrane Collaboration tools Synthesis NR
**Farina (2014) [38]**	6 RCT	*n* = 171 AD MMSE scores 5–29 Age NR	Intervention: exercise Control: no exercise, home safety assessment, daily activity, organized conversation, TAU, support group	**Cognitive function** (ERFC, MMSE, ADAS-cog, ADS-6, BNT, HVLT, CANTAB-Expedio)	Significant positive effect Heterogeneity: 69%	Quality Assessment tool for Quantitative Studies: Moderate-strong

* AD = Alzheimer’s disease; ADAS-Cog = Alzheimer’s Disease Assessment Scale Cognitive section; ADS-6 = Amsterdam Dementia Screening Test 6; BBS = Bergs Balance Score; BNT = Boston Naming Test; CANTAB = The Cambridge Neuropsychological Test Automated Battery; CCT = controlled clinical trial; CDT = Clock drawing test; ERFC = Rapid Evaluation of Cognitive Functions test; FACS = Functional Assessment of Communication Skills; FGA = Functional Gate Assessment; FoG = Freezing of Gait; HVLT = Hopkins Verbal Learning test; H&Y = Hoehn & Yahr; MMSE = Mini-Mental State Exam; PD = Parkinson’s disease; NR = not reported; NRCT = non-RCT; qRCT = quasi-RCT; RCT = randomized controlled trial; SPPB = Short Physical Performance Battery; TAU = treatment as usual; TUG = Timed Up and Go; UPDRS = Unified Parkinson’s Disease Rating Score; 6mWT = 6 minute Walk Test

**All data presented in study did not meet eligibility criteria so only relevant data was extracted.

### Methodological quality assessment

**Fig 2:**
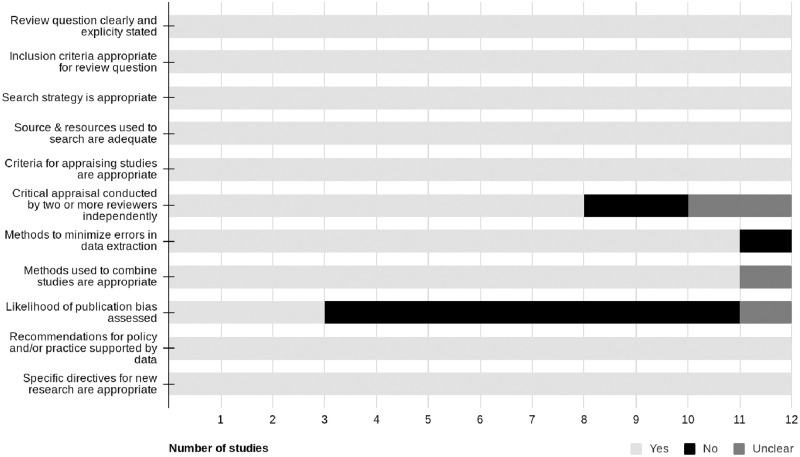
JBI Critical Appraisal Checklist for Systematic Reviews and Research Syntheses. Bar graph highlighting the quality components of included systematic reviews/meta-analyses for reporting methodological quality.

The overall methodological quality of the systematic reviews and meta-analyses are presented in Fig 2. Methodological quality can be considered high due to most components being adequately addressed within the systematic reviews and meta-analyses. The primary explanation for lesser quality was due to publication bias, where assessment was not clearly reported through a visual check of a funnel plot or statistical tests [29–36] or publication bias was not explicitly mentioned [37].

### Disease

The population included in this review was largely homogeneous with most being diagnosed with mild-moderate stages of PD. Three studies included data that observed the effect that active rehabilitation has on the cognitive function of patients with AD [29,34,37] with no other pathologies (i.e., LBD, FTD, CBD) included in this analysis.

### Mood/Behavior

No eligible reviews provided information on symptoms of mood or behavior; therefore, analysis on this area was not possible.

### Vestibular/Ocular (balance)

**Fig 3:**
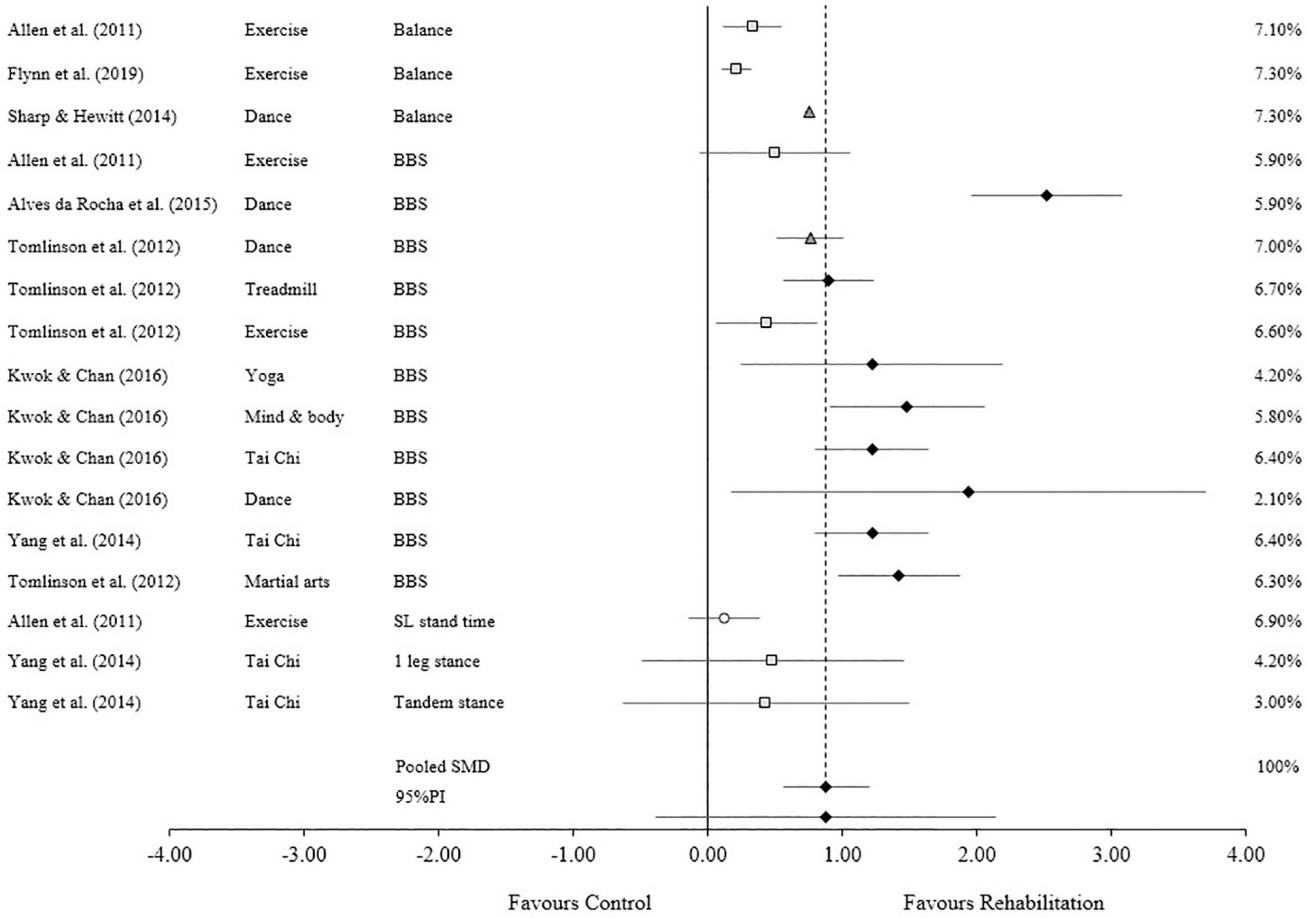
Standardized mean difference (SMD) for vestibular/ocular symptoms (balance). 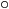
 Trivial effect ■ Small effect ▲ Moderate effect ◆ Large effect. Forest plot to illustrate the standardized mean difference (SMD) (95% confidence intervals) for studies evaluating the effect that active rehabilitation has on measures of balance. BBS: Berg Balance Scale; SL: Single leg.

Assessing the effectiveness of active rehabilitation on vestibular or ocular symptoms (*n* = 7) resulted in the inclusion of balance measures only, primarily using the Berg Balance Scale (BBS) or a component of the BBS, such as single leg or tandem stance. A large pooled SMD was observed (SMD = 0.88, 95% CI 0.56 to 1.21, P <0.001) with a prediction interval ranging from -0.38 to 2.15, albeit the level of heterogeneity was deemed to be considerable (I^2^ = 91.9%, Q = 196.77, P < 0.0001) (Fig 3).

### Motor

**Fig 4:**
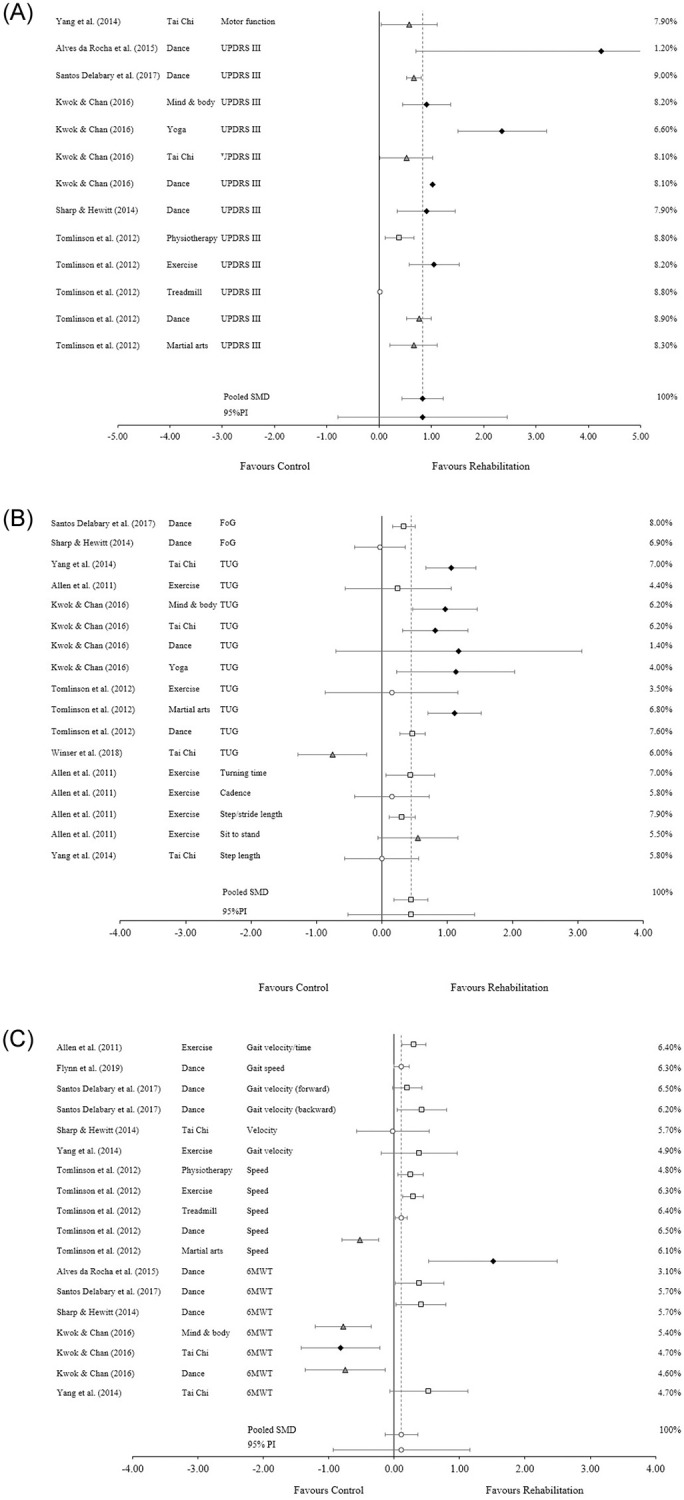
Standardized mean difference (SMD) for motor symptoms. 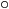
 Trivial effect ■ Small effect ▲ Moderate effect ◆ Large effect. Forest plot to illustrate the standardized mean differences (SMD) (95% confidence intervals) for studies evaluating the effect that active rehabilitation has on measures of motor function. Fig 4A is comprised of measures that observed general motor function. Fig 4B is comprised of measures that observed functional mobility. Fig 4C is comprised of measures that observed gait speed and velocity. FoG: Freezing of gait; TUG: Timed up and go test; UPDRS III: Unified Parkinson’s Disease Rating Score Part III (motor); 6mWT: Six-minute walk test.

Due to most of the included data involving different types of motor function measures, this section was broken into three subsections: motor function health, functional mobility and gait speed/velocity (Fig 4). Motor function (*n* = 6), mainly consisting of UPDRS III outcome scores, observed a large pooled SMD (SMD = 0.83, 95% CI 0.43 to 1.22, P < 0.001). The prediction interval ranged from -0.79 to 2.43 and level of heterogeneity was substantial (I^2^ = 76.8%, Q = 51.75, P < 0.0001) (Fig 4A). Functional mobility (*n* = 7), consisting of measures such as freezing of gait, timed up and go, sit to stand, step length, cadence, sit to stand, and turning time, observed a small SMD (SMD = 0.45, 95% CI 0.19 to 0.71, P = 0.002). The prediction interval ranged from –0.52 to 1.42 and a substantial level of heterogeneity was observed (I^2^ = 74.3%, Q = 62.29, P < 0.0001) (Fig 4B). Gait speed/velocity (n = 8), consisting of measures such as gait velocity/time, speed and the 6-minute walk test, observed a trivial SMD (SMD = 0.11, 95% CI -0.14 to 0.36, P = 0.372). The prediction interval ranged from -0.94 to 1.15 and a substantial level of heterogeneity was observed (I^2^ = 79.8%, Q = 84, P < 0.0001) (Fig 4C).

### Cognitive

**Fig 5:**
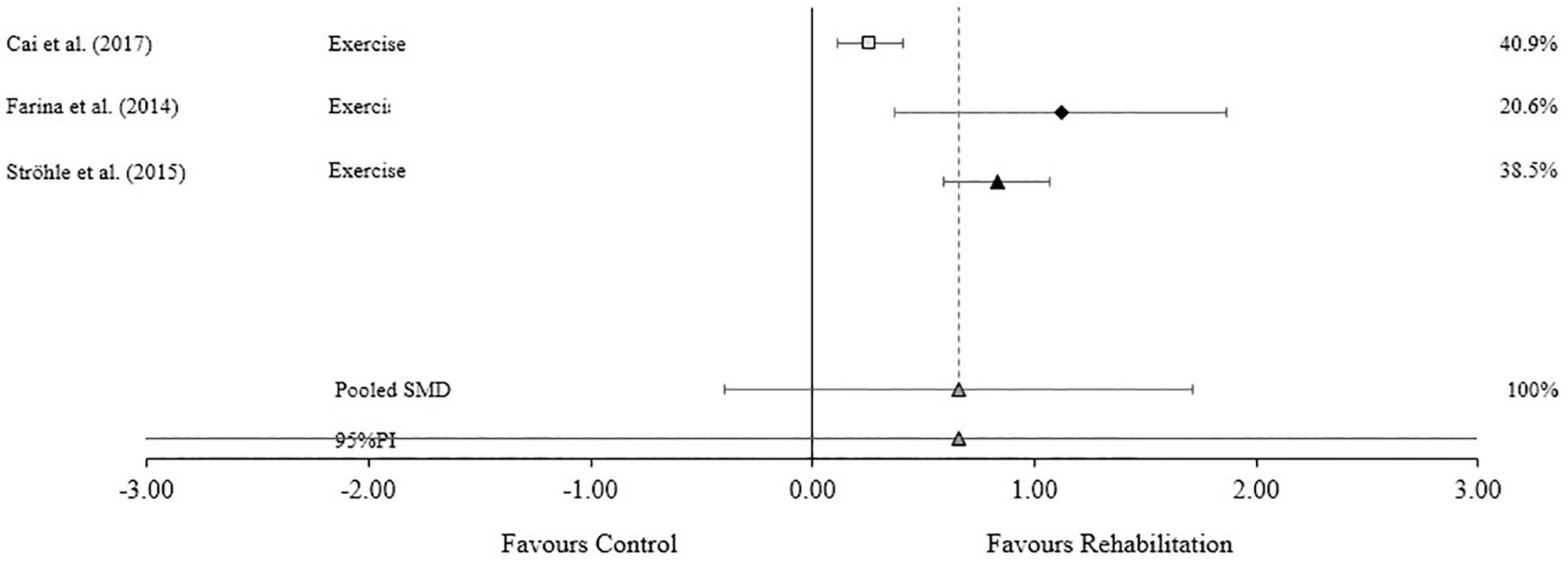
Standardized mean difference (SMD) for global cognitive measures. 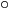
 Trivial effect ■ Small effect ▲ Moderate effect ◆ Large effect. Forest plot to illustrate the standardized mean differences (SMD) (95% confidence intervals) for studies evaluating the effect that active rehabilitation has on measures of cognitive function.

Studies assessing the effectiveness of active rehabilitation on global cognitive symptoms (*n* = 3) result in a moderate pooled SMD (SMD = 0.66, 95% CI -0.40 to 1.71, P = 0.116) but a prediction interval ranging from -1.34 to 2.39. A considerable level of heterogeneity was also evident (I^2^ = 89.4%, Q = 18.79, P < 0.0001) (Fig 5).

## Discussion

The aim of this umbrella review was to evaluate the results of systematic reviews and meta-analyses which report the effect that active rehabilitation has on symptoms associated with CTE that are observed in other tauopathies compared to a control condition. Determined by the size and consistency of the measured effect as well as the quality of the evidence, this study found that active rehabilitation has a large pooled effect on balance and motor function, a moderate pooled effect on cognitive function and a small pooled effect on mobility in populations suffering from tauopathies. Results should be interpreted with caution as all measures demonstrated substantial to considerable levels of heterogeneity and wide 95%PI; however, when considering the SMD, 95%CI, and 95%PI, there is little to no likelihood of a negative or null effect. This provides support for the use of active rehabilitation as a management tool for symptoms associated with tauopathies. This has addressed a gap in the evidence regarding potential intervention strategies for CTE and provides a basis for the use of active rehabilitation in future CTE research.

### Quality

The methodological quality of the systematic reviews or meta-analyses included was found to be high, with a lack of insight into publication bias being the only common error (Table 2).

The quality of evidence gathered to analyze the effect that active rehabilitation has on measures of balance was inconclusive. Multiple studies did not provide necessary information, with other studies not providing specific information that the authors could extract [31,34,36,38]. When these factors were reported, the quality was inconsistent: i) three studies provided good to strong levels of quality [29,31,39] with missing components largely concerning intention to treat and blinding; these factors are difficult to address with studies evaluating the effect of an exercise program, ii) two studies demonstrated weak levels of quality [29,32], reporting issues of selection bias, blinding and global rating, and iii) one study [39] noted a possibility of publication bias due to studies including a small sample and large positive effects.

When evaluating the quality of evidence for the analysis of motor function: i) five studies did not provide specific information that could be effectively extracted [31,33,34,36,38], ii) of those studies which reported quality evidence, most reported high quality of evidence [31,37,39]; common issues included selection bias, blinding, and global rating.

The quality of evidence from studies looking at cognitive function was moderate to strong [30,40]; however, there were potential sources of bias that were not clearly reported by the primary studies. This included allocation concealment, rating of biometric quality and selective reporting. Blinding was again a commonly noted issue. There was no evidence of publication bias.

While the findings are promising, the assessment of the quality of evidence of the meta-analyses included in this review calls for caution.

### Efficacy

While not a core clinical feature, motor impairment is a supportive feature noted in suspected CTE. As seen in Table 1, potential symptoms can include Parkinsonism (gait disturbances, bradykinesia, etc.), muscle rigidity, muscle tremors, and vestibular/ocular impairment (balance, dizziness, double vision, etc.) [5,26]. Evidence suggests that active rehabilitation has beneficial effects on vestibular/ocular symptoms. Specifically, this umbrella review found a positive effect on balance in patients suffering from mild to moderate PD, with only one study including participants with severe levels of PD [38]. Despite an observed large effect, it should be noted that the majority of data points that provided a large effect came from the same study [32] and had wide confidence intervals. In addition, heterogeneity was considerable; however, clinicians can still expect to see small to large improvements in balance based on the 95%CI and 95%PI. This effect was observed regardless of the type of intervention prescribed, one of the likely contributors of a considerable amount of heterogeneity. Reviews included interventions such as tai chi [32,38], yoga [32], dance [29,32,34,36], general exercise (aerobic, resistance training, combination) [31,36,39], martial arts [36] and treadmill training [36]. Duration, frequency and intensity also varied across the studies. The most common outcome measure used was the BBS. Three [38,39] of the four studies which used a single component of the BBS, the single-leg stance, reported a null or negative effect which suggests the interventions used might have a more rounded effect than that reflected in a single measure. Still, evidence indicates that active rehabilitation will produce a positive effect on measures of balance in populations suffering from tau pathology; however, the expected effect size is less certain due to the considerable level of heterogeneity and a wide 95%PI.

This review indicates that active rehabilitation has a notable positive effect on motor function and mobility; though it is worth noting that the only meta-analyses included in this review that assessed motor function and mobility included patients with mild to severe PD. Although the 95%PI indicates there is a small chance that a future study may produce null or negative results, the pooled SMD suggests a likely improvement in UPDRS III scores, a scale that measures the motor function abilities of those suffering from PD. Interestingly, the more successful interventions were mostly those that fall under the category of mind and body, including yoga, tai chi, dance and martial arts. These interventions put a great deal of focus on mind-body coordination, spatial awareness [32,37,38] and smooth movements [37]. The variation in intervention mode delivered (treadmill, tai chi, strength training, etc.), exercise prescriptions (frequency, intensity, duration), and the large range of sample sizes included likely contributed to the substantial level of heterogeneity. Regardless, this review illustrates that active rehabilitation produces a positive effect on motor function symptoms; however, the size of the effect is uncertain due to the wide 95%PI and substantial level of heterogeneity.

Clinicians can also expect small to moderate improvements in timed up and go (TUG) tests, a commonly used measure of functional mobility. There were multiple data points that observed a large effect; however, these were largely from the same study [32]. Only two other studies observed a large effect [36,38] with the rest of the data reporting trivial [36] to small [36,39] positive effects, and one moderate negative effect [37]. Regardless, the 95%CI and 95%PI which illustrate a likely small positive effect. Other measures, such as freezing of gait and gait analysis, are inconsistent with some showing a null effect [34,38,39] and others producing a small to moderate positive effect [33,38,39]. Again, the presence of multiple intervention programs and prescriptions contributed to a substantial level of heterogeneity. No intervention type seemed to be more successful than others. Evidence from this review suggests that active rehabilitation has a positive effect on measures of functional mobility in tau pathology; however, the expected effect size varies as indicated by the varying pooled effect sizes, wide 95%PI measures and substantial levels of heterogeneity.

The effect that active rehabilitation has on symptoms of gait speed/velocity is inconclusive due to the observed trivial effect along with both 95%PI and 95%CI showing equal likelihood of negative, null/trivial, and positive effects. While many of the reviews showed a small to moderate positive effect, those that produced negative results had a larger effect [32,36]. Only one study produced a large positive effect [31], accompanied by wide a 95%CI. Heterogeneity was substantial, likely from the various interventions, prescriptions and outcome measures used. Dance and Tai Chi produced both positive and negative effects, with exercise and physiotherapy producing modest improvements. This review did not provide conclusive evidence on the effect that active rehabilitation has on symptoms of gait speed/velocity in patients with tau pathology.

Cognitive dysfunction is one of the core clinical features for identifying potential CTE pathology [41], with executive function, episodic memory, mental flexibility, semantic verbal fluency, and attention and processing speed being some of the more notable impairments. Evidence suggests that active rehabilitation has a moderate effect on cognitive symptoms in AD populations, the only population included in the meta-analyses of cognitive function assessed in this umbrella review. The lack of inclusion of other tauopathies and the small number of studies assessed suggests findings should be interpreted with caution when extrapolating to other populations. Given the small number of studies, the 95%PI offers little information; however, preliminary findings are positive. Despite the 95%CI suggesting a small chance of null or negative findings, two [30,35] of the three included studies offer small to medium effects. The third study [40] found a large effect with a larger number of studies and participants included. Heterogeneity in this analysis was considerable and is likely explained by disease severity and intervention prescriptions. All studies included substantial to considerable levels of heterogeneity. Despite all studies including AD patients, the study with the lowest level of heterogeneity had a smaller sample size with a smaller range of severity scores [35] meaning greater certainty can be ascertained from this analysis (moderate SMD). This study [35] had less variability with its intervention prescription durations as well, something also noted in Farina et al [40] which had slightly less variability with the duration and frequency of the interventions prescribed compared to Cai et al [30]. The variability of outcome measures used could also introduce high levels of heterogeneity, with nine different tools included. Despite a wide 95%PI and considerable levels of heterogeneity creating uncertainty in the expected size of the effect, the evidence suggests that patients with tau pathology will experience a positive effect on cognitive symptoms with active rehabilitation.

Despite this umbrella review observing the effect of active rehabilitation techniques across multiple tauopathies, applicability to CTE is supported by the underpinning physiological mechanisms that active rehabilitation may elicit. While the mechanisms and areas affected may differ between tauopathies, the progressive neural degeneration and associated clinical symptoms are attributed to synaptic dysfunction and impairments to neural connectivity which accumulated p-tau creates. With no intervention, the process leads to neural cell death and subsequent atrophy of affected regions [3,4]. Exercise can promote neurogenesis and improve cerebral blood flow [18–20]. There are models which present exercise as a disease-modifying intervention for patients with tauopathies, offering a potential system which can also reverse and prevent further damage due to the presence of tauopathies. One mechanism proposes the ability exercise has to target factors which regulate pathogenic tau production and accumulation [42,43]. Another mechanism discusses how exercise is able to enhance cellular and molecular mechanisms that support a healthy neural environment, such as autophagic (impaired by pathogenic p-tau) and anti-inflammatory systems [43] which can counteract pro-inflammation-related neuronal damage triggered by pathogenic p-tau.

When considering the consistency of positive findings and reported pooled effect sizes across systematic reviews or meta-analyses that investigate the impact of active rehabilitation on various tauopathies, this umbrella review has provided evidence to support the use of active rehabilitation as a management tool for suspected CTE—a condition where currently no experimental intervention studies have been published. Despite the heterogeneity observed across this umbrella review, including different tauopathies, different levels of disease severity, different intervention modes and different outcomes, the reported effects are largely positive. Only the effect on measures of gait speed/variability remains inconclusive with the likelihood of a positive, null or negative seemingly equal. The effectiveness of active rehabilitation on measures of functional mobility appears to depend on the assessment utilized. A positive effect is more consistent in studies that utilize the TUG test. The overall size of the effect on measures of functional mobility is small as indicated by the pooled effect. The effect on symptoms of motor and cognitive function should be interpreted with caution. The calculated confidence and prediction intervals suggest a small likelihood of null or negative findings; however, the pooled moderate and large effects suggest a generally beneficial effect. Measures of balance provide the strongest and most consistent positive effect in this review, accompanied by a large pooled SMD. The degree of effectiveness for motor function, cognitive function and balance remains to be determined, as indicated by large variability in 95% confidence and prediction intervals. Future research should consider sub-analysis of factors such as disease, disease severity and intervention to address high levels of heterogeneity and determine more precise effect size estimates. This review has provided preliminary evidence to support the use of active rehabilitation as a therapeutic option for management of clinical symptoms and health outcomes of common tauopathies, including CTE.

### Future research

There are two gaps that emerged within this review. The first is the lack of systematic reviews or meta-analyses addressing the effect that active rehabilitation has on tauopathies other than PD. This includes AD, LBD, FTD, CBD. The other gap is the effect that active rehabilitation has on mood/behavior symptoms of tauopathies, a critical gap that is needed to better understand the potential use for CTE patients. Mood and behavior symptoms make up the other two core clinical features for identifying suspected CTE [41]. A major cause of these gaps is the methodology employed in primary level studies and the eligibility criteria employed at the secondary levels of research (systematic reviews and meta-analyses). More information on AD, LBD, and mood/behavior impairments would have been included in this analysis had inactive/treatment as usual control groups and extractable data been presented. Indeed, more than 82 systematic reviews and meta-analyses were excluded at this stage in the screening process which included both AD and LBD populations as well as cognitive, motor and mood/behavior outcome measures. Due to the increased heterogeneity and variability that is expected in an umbrella review, a non-active rehabilitation control group is necessary to effectively evaluate whether active rehabilitation affects symptoms associated with tauopathies. The other cause of these gaps is the overall lack of studies observing the effect that active rehabilitation has on populations suffering from CBD and FTD. Case studies have been performed and note improvements in balance, walking, gait, executive function, attention, and depressive symptoms [44–46]; however, no further research could be identified. The lack of studies observing CBD can be explained by the rarity of the disease which has caused a general lack of knowledge regarding identification and treatment options [47]. FTD is also in its research infancy with most efforts going towards identification techniques [48].

## Conclusion

While available research is limited to a few types of tauopathies and detailed information on mood/behavior symptoms is scarce, the results of this umbrella review report positive effects on measures of balance, motor function and functional mobility, and cognitive function in the management of tauopathies. Within these broad areas specific activities have emerged as potential candidates for inclusion in active rehabilitation programs for CTE patients; these include tai chi, yoga, dance, general exercise (aerobic, resistance training, combination), martial arts, treadmill training and formal physiotherapy. There is further evidence not included in this review to support the use of active rehabilitation in other tauopathies (LBD, CBD and FTD) and for mood and behavior symptoms, but more research is needed to better support this theory. Regardless, this review provides preliminary evidence to support future research which seeks to investigate the effect that active rehabilitation has on patients with suspected CTE.

## Supporting information

S1 Data (PDF)

S2 Data (PDF)

S3 Data (PDF)
