# Supplementary material for: The effects of active rehabilitation on symptoms associated with tau pathology: An umbrella review. Implications for chronic traumatic encephalopathy symptom management
Source: PLoS One. 2022 Jul 21;17(7):e0271213. doi: 10.1371/journal.pone.0271213 (PMC9302815; doi:10.1371/journal.pone.0271213)
Supplement: S1 Data — (PDF) [file pone.0271213.s001.pdf]

Title	Author/Year		Objectives	Participants (Characteristics/Total No/Sex)			Neuro disease	Setting/Context/Stage of disease	Description of interventions/phenomena of interest	Search details	Sources searched	Range (yrs) of included studies	Number of studies included	Types of studies included	Country of origin of included studies	Appraisal/Appraisal Instruments/Appraisal Rating	Method of analysis	Outcome assessed (relevant to study)	Significance/direction	Heterogeneity (I^2)
Balance and Falls in Parkinson's Disease: A Meta-analysis of the Effect of Exercise and Motor Training	Allen et al.	####	To examine if exercise has an effect on balance-related activity and/or falls in people with PD. Meta-regression used to evaluate what effect that total dose of exercise and presence of highly challenging balance activity has.	Synthesis NR	747	NR	PD	Stages of PD: mild - moderate	Intervention:exercise (aerobic, resistance, Tai Chi, dance)<br>Control: no intervention, TAU, education classes, flexibility exercise	Not available.	MEDLINE, EMBASE, AMED, PsycINFO, Cochrane Central Register of Controlled Trials, CINAHL. Supplemented with searches of Physiotherapy Evidence Database and search of citations.	1996-2009	19	RCT, quasi-randomized controlled trials	NR	Cochrane risk of bias tool<br>Mod-high quality: 7<br>Insufficient info: 8<br>Eggers: 1.69<br>Funnel plot: evidence of publication bias	Random-effects meta-analysis<br>Hedge's g (SMD) calculated using pre-/post-mean and SD	BBS, single leg stand time, tandem stance, TUG, sit to stand time, turning time, step length, cadence, gait time, gait velocity	Significant positive effect on balance. Non-significant positive effect on functional mobility and gait.	Balance: 0-72%<br>Turning time: 0%<br>Functional mobility: 0-37%<br>Gait: 6%<br>(Dependent on outcome measure)
Complementary physical therapies for movement disorders in Parkinson's disease: a systematic review	Alves Da Rocha et al.	####	Evaluate the effect that complementary physical therapy has on motor disabilities in people with PD.	NR	NR	NR	PD	NR	Intervention: dance<br>Control: no intervention	Parkinson's disease, rehabilitation, non-conventional physical therapies, alternative therapies, complementary physical therapies, exercise therapy, socio environmental therapy	Medline, Embase, Cinahl, The Cochrane Library, Pedro	2006-2014	2	RCT	NR	PEDro scale<br><br>Good: 1<br>Fair: 1	Descriptive analysis with interventions and variables<br>Meta-analysis of RCTs conducted when possible	BBS, UPDRS III, 6mWT	Positive effect on for gait, balance and motor function	Balance: NA<br>Motor function: 97%<br>Gait: 91%
Dance as an intervention for people with Parkinson's disease: A systematic review and meta-analysis	Sharp et al.	####	Evaluate how effecticve dance is compared to no intervention and other exercise interventions.	Mean age: 66.6, 69.9	NR	NR	PD	Mean Hoehn and Yahr stage: 2.1, 2.6	Intervention: any form of dance<br>Control: no intervention, other exercise interventions	Parkinson*, danc*, Random*	AMED, BNI, CINAHL, EMBASE, HBE, HMIC, MEDLINE, PscyhINFO, Cochrane Central Registeer of COntrolled Trials, Web of Knowledge, OTseeker, PEDro, SpeechBITE, PsychBITE, Rehabdata, LILACS, IMEMR, MedCarib, DART, Electronic Thesis online service, Networked digital library of Theses, Cochrane controlled trials register, clinicaltrials.gov, National institute for health research, Trialsjournal.com .	2007-2013	2	RCT	NR	Cochrane Collaborations risk of bias assessment tool<br>Individual reports not available.	Mean difference w/95% CI were calculated<br>Fixed effects invariance model	BBS, UPDRS III, FoG, 6mWT, gait velocity	Significant positive effect on motor function, balance, gait velocity. No effect on functional mobility.	Balance: 0%<br>Motor function: 0%<br>Functional mobility: 0%<br>Gait: 0-45%<br>(Dependent on outcome measure)
Doe Tai Chi improve balance and reduce falls incidence in neurological disorders? A systematic review and meta-analysis	Winser et al.	####	Determine whether Tai Chi can improve balance and reduce fall rate in people with neurological disorders, comparing to no intervention and other active treatments.	NR	96	NR	PD	NR	Intervention: Tai Chi<br>Control: no intervention, other active treatments	Specifics NR	AMED, Embase, Web of Science, SCOPUS, EBSCO, and Medline	2008-2015	2	RCT	Hong Kong, China, Republic of Korea, United States, Korea, Taipei, Taiwan	PEDro: high<br><br>GRADE: high	Pooled estimate of treatment effect, 95% CI OR or WMD<br>Random or fixed effects based on heterogeneity	TUG	Significant effect	NR
Drug and exercise treatment of Alzheimer's disease and mild cognitive impairment: a systematic review and meta-analysis of effects on cognition in randomized controlled trials	Ströhle et al.	####	To directly compare drug therapy and exercise treatments for people with MCI or AD.	NR	119	NR	AD	MMSE scores:<br>13-22	Intervention: exercise treatment<br>Control: TAU, daily organized activities, home safety assessment sessions	Combinations of: RCT, mild cognitiv*, Alzheimer's disease, physical activity, (drug)	The Cochrane Library, EBSCO, OVID, Web of Science, and FDA.	1996-2013	4	RCT	NR	Cochrane Collaboration's tool for assessing risk of bias<br>Synthesis NR	SMCR, pooled ES	Cognitive function	Moderate to strong effect.	61.60%
Effect of exercise on cognitive function in chronic disease patients: a meta-analysis and systematic review of randomised controlled trials	Cai et al.	####	Assess the effect of exercise on cognitive function in people with chronic diseases.	Age range: 72.4-81.8	958	NR	AD	MMSE scores: 5.8-22	Intervention: exercise (aerobic, resistance, combined)<br>Control: no exercise	Combinations of: Cognition, cognitive function, MMSE, exercise, mscule stretching exercises, resistance training, running or swimming, walking, cycling, physical activity, aerobic, yoga, tai chi, qigong, RCT	PubMed, Web of Science, Embase, the Cochrane Liibrary, CINAHL, PscyINFO, CNKI, WanFang Datam and VIP.	NR	13	RCT	NR	Downs and Black Quality Index: mean score 22.05<br>5: good<br>7: moderate<br>1: poor	SMD, 95% CI<br>Random effects model	Global cognitive function	Positive overall random effect on cognitive function	AD: 77%
Effects of dance practice on functional mobility, motor symptoms, and quality of life in people with Parkinson's disease: a systematic review with meta-analysis	Santos Delebary et al.	####	Deteremine the effects that dance classes have on mobility, motor symptoms, and QoL in people with PD when compared to other interventions or no intervention.	Age range: 66.5+/-2.8 to 69.3+/-1.9	83	NR	PD	H&Y stage range 1-4	Intervention: dance classes<br>Control: no dance classes, no intervention	Parkinson's disease, dancing, and specific filter for randomized controlled trials	MEDLINE, LILACS, SciELO, Cochrane and PsychINFO.	2007-2015	2	RCT	NR	Cochrane criteria<br>Synthesis NR	Pooled effect size, WMD, SD<br>Fixed or random effects depending on heterogeneity	UPDRS III, FoG, 6mWT, gait velocity – forward, backward	Significant positive effect on motor function. Non-significant positive effect on gait and functional mobility.	0% (for data included)
Effects of mind-body exercises on the physiological and psychosocial well-being of ondividuals with Parkinson's disease: A systematic review and meta-analysis	Kwok et al.	####	Evaluate the effect of mind-body exercises on the physiological and psychological outcomes for people with PD.	Mean age range: 60.8-74.9	NR	NR	PD	Mild - moderate	Intervention: mind-body exercise treatment<br>Control: no intervention, placebo, waitlist, usual care, non-exercise control	Parkinson, Mind body, Tai Chi, Qigong, Yoga, Dance, Pilates	EMBASE, Ovid Medline, Psych Info, Cochrane Library	NR	10<br>RCT: 6<br>CCT: 4	RCT, CCT	United States, Aisa, Europe	EPHPP<br>1: strong<br>5: moderate<br>4: weak	Means & std dev for absolute change<br>Random effects if high heterogeneity	BBS, UPDRS III, TUG, 6mWT	Large significant effect on motor symptoms, balance and postural instability. Moderate significant effect on functional mobility	Balance: 0%-89%<br>Motor function: 0-60%<br>Functional mobility: NA-95%<br>Gait: NA-0%<br>(Dependent on intervention mode)
Home-based prescribed exercise improves balance-related activities in people with Parkinson's disease and has benefits similar to centre-based exercise: a systematic review	Flynn et al.	####	Determine if home-based exercise can improev balance and QoL in people with PD, and determine if the effects are comparable to centre-based exercise programs.	Mean age: 60-72	1496	NR	PD	Mild-moderate	Intervention: home-based exercise<br>Control: TAU, placebo	Parkinson's disease, physiotherapy, exercise home-based therapy, group-based therapy, supervision, gait, mobility, balance, quality of life, randomised and quasi-randomised	CINAHL, CENTRAL, EMBASE, Physiotherapy Evidence Database	2005-2018	12	RCT, qRCT	NR	PEDro<br>10: good<br>2: fair	Pooled effect size, Hedges g<br>Fixed or random effects depending on heterogeneity<br>Pairwise & subgroup analyses for dose/supervision effect analysis	SPPB, BBS, miniBESTest, time taken to walk, preferred gait speed, fast gait speed, TUG, FGA, 180 deg. turn test	Positive effect on balance and gait speed	Balance: 0%<br>Gait speed: 0%
Physiotherapy intervention in Parkinson's disease: a systematicc review and meta-analysis	Tomlinson et al.	####	Provide an overall assessment of the effect physiotherapy has on people with PD.	Range: 65-69	1570	NR	PD	Range of H&Y: 2.1-2.6	Intervention: physiotherapy, exercise, treadmill, dance, martial arts<br>Control: no intervention, placebo	Physiotherapy, physical therapy, exercise, rehabilitation, Parkinson, Parkinson's disease, parkinsonism	Medline, Embase, Cumulative Index to Nursing and Allied Health Literature, Web of Science, Allied and Complimentary Medicine Database, REHABDATA, REHADAT, GEROLIT, Latin American and Caribbean Health Sciences Literature, MedCarib, Index medicus for the Eastern Mediterranean region, conference databases, and trial registries. Specific journals were also hand searched.	1998-2012	20	RCT	NR	Synthesis NR	WMD, 95% CI	BBS, UPDRS III, gait speed, TUG	Significant positive effect on balance, gait and motor function.	Balance: NA-75%<br>Motor function: 0%-87%<br>Functional mobility: 0%-48%<br>Gait: 0%-34%<br>(Dependent on intervention mode)
Tai Chi for Improvement of Motor Function, Balance, and Gait in Parkinson's Disease: A Systematic Review and Meta-Analysis	Yang et al.	####	Evaluate the efficacy of Tai Chi on people with PD.	Range: 63-69	190	NR	PD	Range of H&Y: 1.5-4	Intervention: Tai Chi<br>Control: placebo, no intervention, other therapies	Parkinson's disease, Parkinson, Tai Chi, taiji, shadowboxing	PubMed, EMBASE, Cochrane Library, China Knowledge Integrated Database, Wejpu Database for Chinese Technical Perioidicals, Wan Fang Data. ProQuest Dissertations and Cinese dissertation database, WHO International Clinical Trials Registry Platform	2008-2014	5<br>RCT: 4<br>NRCT: 1	RCT, NRCT	China, Korea, US	Cochrane Collaboration tools<br>Synthesis NR	Pooled estimate of efficacy, SMD, 95% CI	BBS, 1 leg stance, tandem stance, UPDRS III, TUG, gait velocity, 6mWT	Significant positive effect on balance, motor function and functional mobility. Insufficient evidence of effect on gait.	Balance: 0-68%<br>Motor function: 57%<br>Functional mobility: 0%<br>Gait: 0%<br>(Dependent on outcome measure)
The effect of exercise interventions on cognitive outcome in Alzheimer's disease: a systematic review	Farina et al.	####	To assess how effective exercise is in attenuating cognitive decline in people with AD.	NR	NR	171	AD	MMSE range: 5-29	Intervention: exercise<br>Control: no exercise, home safety assessment, daily activity, organized conversation, TAU, support group	Exercise, physical, trianing, walk*, danc*, movement, Alzheimer's, dementia, intervention, program	PubMed, Science Direct, Web of Knowledge, PsychINFO	2004-2012	6	RCT	NR	Quality Assessment tool for Quantitative Studies: moderate-strong<br>Funnel plot for publication bias: none found	Effect size, 95% CI	ERFC, MMSE, ADAS-cog, ADS-6, BNT, HVLT, CANTAB-Expedio	Significant positive effect	69%
