# Supplementary material for: The effects of active rehabilitation on symptoms associated with tau pathology: An umbrella review. Implications for chronic traumatic encephalopathy symptom management
Source: PLoS One. 2022 Jul 21;17(7):e0271213. doi: 10.1371/journal.pone.0271213 (PMC9302815; doi:10.1371/journal.pone.0271213)
Supplement: S2 Data — (PDF) [file pone.0271213.s002.pdf]

	Measure	Review Author	SMD	95% CI	Lower CI (SMD)	Upper CI (SMD)
	**Balance**					
Exercise and motor training	Balance	Allen et al.	0.33	0.22	0.11	0.55
Exercise vs. no exercise	Balance	Flynn et al.	0.21	0.11	0.10	0.32
Dance vs. no intervention	Balance	Sharp et al.	0.76	0.02	0.72	0.76
Exercise and motor training	BBS	Allen et al.	0.50	0.56	-0.06	1.06
Dance vs. no intervention	BBS	Alves da Rocha et al.	2.52	0.56	2.03	3.15
Dance vs. no intervention	BBS	Tomlinson et al.	0.76	0.25	0.53	1.02
Treadmill vs. no intervention	BBS	Tomlinson et al.	0.90	0.33	0.59	1.25
Exercise vs. no intervention	BBS	Tomlinson et al.	0.44	0.38	0.06	0.81
Yoga vs. no intervention	BBS	Kwok et al.	1.22	0.98	0.25	2.20
Meta (Mind body vs no	BBS	Kwok et al.	1.48	0.58	0.91	2.06
Tai chi vs. no intervention	BBS	Kwok et al.	1.22	0.43	0.80	1.65
Dance vs. no intervention	BBS	Kwok et al.	1.94	1.77	0.18	3.71
Tai Chi vs. no intervention	BBS	Yang et al.	1.22	0.43	0.80	1.65
Martial arts vs no	BBS	Tomlinson et al.	1.42	0.46	1.01	1.93
Exercise and motor training	SL stand time	Allen et al.	0.12	0.27	-0.15	0.38
Tai Chi vs. no intervention	1 leg stance	Yang et al.	0.48	0.98	-0.49	1.46
Tai Chi vs. no intervention	Tandem stance	Yang et al.	0.43	1.07	-0.64	1.50
		Pooled Effect	0.88	0.33	0.56	1.21
		Prediction interval	0.88	1.27	-0.38	2.15
	**Gait Speed/Velocity**					
Exercise and motor training	Gait velocity/time	Allen et al.	0.27	0.18	0.09	0.44
Dance vs. no intervention	Gait speed	Flynn et al.	0.30	0.19	0.12	0.49
Dance vs. no intervention	Gait velocity (forward)	Delebary et al.	0.11	0.11	0.00	0.23
Dance vs. no intervention	Gait velocity (backward)	Delebary et al.	0.20	0.23	-0.02	0.43
Tai Chi vs. no intervention	Velocity	Sharp et al.	0.42	0.38	0.04	0.80
Exercise vs. no exercise	Gait velocity	Yang et al.	-0.02	0.56	-0.58	0.54
General physio vs. no	Speed	Tomlinson et al.	0.38	0.58	-0.20	0.96
Exercise vs. no intervention	Speed	Tomlinson et al.	0.25	0.19	0.06	0.45
Treadmill vs. no intervention	Speed	Tomlinson et al.	0.29	0.16	0.13	0.45
Dance vs. no intervention	Speed	Tomlinson et al.	0.11	0.09	0.02	0.20
Martial arts vs no	Speed	Tomlinson et al.	-0.52	-0.28	-0.26	-0.81
Dance vs. no intervention	6MWT	Alves da Rocha et al.	1.51	0.99	0.53	2.50
Dance vs. no intervention	6MWT	Delebary et al.	0.39	0.37	0.01	0.76
Dance vs. no intervention	6MWT	Sharp et al.	0.41	0.38	0.03	0.79
Meta (Mind body vs no	6MWT	Kwok et al.	-0.78	0.43	-1.21	-0.35
Tai chi vs. no intervention	6MWT	Kwok et al.	-0.82	0.61	-1.43	-0.22
Dance vs. no intervention	6MWT	Kwok et al.	-0.75	0.61	-1.36	-0.14

Tai Chi vs. no intervention	6MWT	Yang et al.	0.53	0.60	-0.07	1.12
		Pooled Effect	0.11	0.25	-0.14	0.36
		Prediction interval	0.11	1.05	-0.94	1.15
	Functional Mobility					
Dance vs. no intervention	FoG	Delebary et al.	0.34	0.17	0.17	0.51
Dance vs. no intervention	FoG	Sharp et al.	-0.03	0.39	-0.42	0.36
Exercise and motor training	TUG	Yang et al.	1.06	0.38	0.68	1.44
Exercise and motor training	TUG	Allen et al.	0.25	0.81	-0.59	1.03
Exercise and motor training	TUG	Kwok et al.	0.97	0.50	0.48	1.47
Exercise and motor training	TUG	Kwok et al.	0.82	0.50	0.32	1.32
Tai Chi vs. no intervention	TUG	Kwok et al.	1.18	1.88	-0.70	3.07
Tai Chi vs. no intervention	TUG	Kwok et al.	1.13	0.90	0.23	2.03
Tai Chi vs. no treatment for	TUG	Tomlinson et al.	0.15	1.02	-0.87	1.17
Exercise and motor training	TUG	Tomlinson et al.	1.12	0.41	0.72	1.53
Meta (Mind body vs no	TUG	Tomlinson et al.	0.47	0.20	0.28	0.67
Tai chi vs. no intervention	TUG	Winser et al.	-0.76	0.53	-1.29	-0.23
Dance vs. no intervention	Turning time	Allen et al.	0.44	0.37	0.07	0.81
Yoga vs. no intervention	Cadence	Allen et al.	0.16	0.57	-0.75	0.40
Exercise vs. no intervention	Step/stride length	Allen et al.	0.31	0.20	0.12	0.51
Martial arts vs no	Sit to stand	Allen et al.	0.55	0.61	-0.06	1.16
Dance vs. no intervention	Step length	Yang et al.	0.00	0.57	-0.57	0.56
		Pooled Effect	0.45	0.26	0.19	0.71
		Prediction interval	0.45	0.97	-0.52	1.42
	Motor Function					
Tai Chi vs. no intervention	Motor function	Yang et al.	0.57	0.54	0.04	1.11
Dance vs. no intervention	UPDRS III	Alves da Rocha et al.	4.24	3.54	0.70	7.78
Dance vs. no intervention	UPDRS III	Delebary et al.	0.66	0.14	0.52	0.80
Meta (Mind body vs no	UPDRS III	Kwok et al.	0.91	0.46	0.45	1.37
Yoga vs. no intervention	UPDRS III	Kwok et al.	2.35	0.85	1.50	3.20
Tai chi vs. no intervention	UPDRS III	Kwok et al.	0.52	0.51	0.02	1.03
Dance vs. no intervention	UPDRS III	Kwok et al.	1.02	0.00	0.53	0.53
Dance vs. no intervention	UPDRS III	Sharp et al.	0.90	0.55	0.35	1.45
General phsyio vs. no	UPDRS III	Tomlinson et al.	0.39	0.27	0.12	0.66
Exercise vs. no intervention	UPDRS III	Tomlinson et al.	1.05	0.48	0.32	1.28
Treadmill vs. no intervention	UPDRS III	Tomlinson et al.	0.01	0.03	-0.02	0.04
Dance vs. no intervention	UPDRS III	Tomlinson et al.	0.76	0.23	0.53	0.99
Martial arts vs no	UPDRS III	Tomlinson et al.	0.66	0.45	0.21	1.11
		Pooled Effect	0.83	0.40	0.43	1.22
		Prediction interval	0.83	1.62	-0.79	2.44
	Cognitive Function					

Exercise vs. no exercise	Cognition	Cai et al.	0.26	0.15	0.11	0.41
Exercise vs. no exercise	Global cognition	Farina et al.	1.12	0.75	0.37	1.87
Exercise vs. no exercise	Cognition	Ströhle et al.	0.83	0.24	0.59	1.07
		Pooled Effect	0.66	1.05	-0.40	1.71
		Prediction interval	0.66	5.66	-5.00	6.32
